# Green Synthesis of Gold Nanoparticles Using Carrageenan Oligosaccharide and Their In Vitro Antitumor Activity

**DOI:** 10.3390/md16080277

**Published:** 2018-08-07

**Authors:** Xiangyan Chen, Xia Zhao, Yanyun Gao, Jiaqi Yin, Mingyue Bai, Fahe Wang

**Affiliations:** 1 Shandong Provincial Key laboratory of Glycoscience and Glycoengineering, and Key Laboratory of Marine Drugs, Ministry of Education, School of Medicine and Pharmacy, Ocean University of China, Qingdao 266003, China; cxyqin@126.com (X.C.); gaoyanyun1991@126.com (Y.G.); 18669859101@163.com (J.Y.); baimyue@126.com (M.B.); 2 Laboratory for Marine Drugs and Bioproducts, Qingdao National Laboratory for Marine Science and Technology, Qingdao 266237, China; 3 State Key Laboratory of Bioactive Seaweed Substances, Qingdao Brightmoon Seaweed Group Co., Ltd., Qingdao 266400, China; 86612019@163.com

**Keywords:** carrageenan oligosaccharide, gold nanoparticles, response surface methodology, green synthesis, anti-tumor activity

## Abstract

Gold nanoparticles (AuNPs) have been widely used in catalysis, photothermal therapy, and targeted drug delivery. Carrageenan oligosaccharide (CAO) derived from marine red algae was used as a reducing and capping agent to obtain AuNPs by an eco-friendly, efficient, and simple synthetic route for the first time. The synthetic conditions of AuNPs were optimized by response surface methodology (RSM), and the CAO-AuNPs obtained were demonstrated to be ellipsoidal, stable and crystalline by means of transmission electron microscopy (TEM), scanning electron microscopy (SEM) and X-ray diffraction (XRD). The CAO-AuNPs showed localized surface plasmon resonance (LSPR) oscillation at about 530 nm with a mean diameter of 35 ± 8 nm. The zeta potential of CAO-AuNPs was around −20 mV, which was related to the negatively charged CAO around AuNPs. The CAO-AuNPs exhibited significant cytotoxic activities to HCT-116 and MDA-MB-231 cells, which could be a promising nanomaterial for drug delivery.

## 1. Introduction

Gold nanoparticles (AuNPs) are commonly used nanoparticles which have been widely employed in photoacoustic imaging, catalysis, photothermal therapy and targeted drug delivery [1,2,3,4]. The chemical reduction method is the most widely used to prepare AuNPs [5]; however, some chemical organic compounds including thiol and sodium borohydride are toxic to the environment [6,7]. In the last few decades, the green synthesis of AuNPs has attracted extensive attention due to its use of natural products as a reductant which are eco-friendly, non-toxic and biocompatible [8,9,10]. It has been reported that natural extracts from microorganisms, plants and other material can be used to prepare AuNPs. However, the active ingredients were not clear and the process was relatively complicated and time-consuming [11,12,13]. It is imperative to find the biopolymers with a defined structure to obtain AuNPs by the green synthesis method [2,10].

Some marine carbohydrates, such as fucoidan, agarose and chitosan oligosaccharide have been reported to prepare AuNPs for biodegradability, abundance, and non-toxicity [2,10,14]. Carrageenan is a sulfated linear polysaccharide extracted from marine red algae and composed of d-galactose residues linked alternately by (1→3)-linked *β*-d-galactopyranose (unit G) and (1→4)-linked *α*-d-galactopyranose (unit D) [15,16]. Carrageenan oligosaccharide (CAO) possesses various pharmacological activities, such as anti-tumor and anti-viral activity, for its small molecular weight, good water solubility, and a certain degree of sulphation [17,18,19]. However, to the best of our knowledge, no studies of CAO in the preparation of AuNPs have been reported. It has been believed that the electrostatic interaction of functional groups of marine carbohydrates, such as the hydrogen bond and sulfate group, plays key roles in the stabilization of AuNPs [2,20].

**Figure 1:**
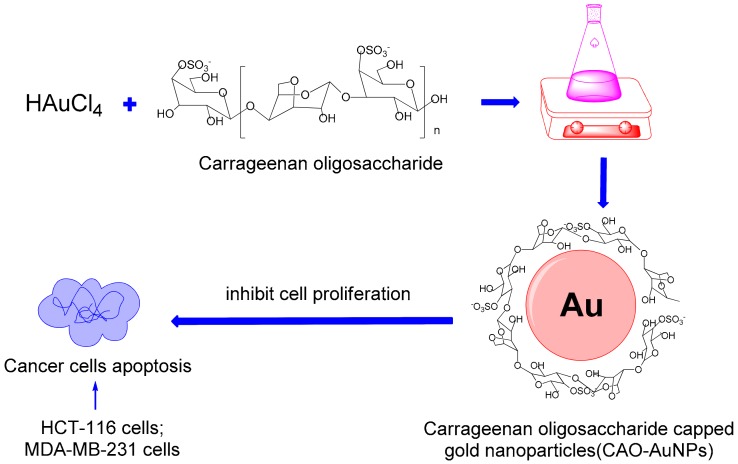
Schematic diagram showing carrageenan oligosaccharide (CAO) reduced Au^3+^ ions and capped gold nanoparticles (AuNPs) and, subsequently, the cytotoxicity of CAO-AuNPs on cancer cells (HCT-116 cells and MDA-MB-231 cells).

Response surface methodology (RSM) is an effective method to determine the effects of multiple factors and relationships among them in order to obtain the optimal level of each factor. RSM possesses various optimization methods and the Box–Behnken design (BBD) is commonly used for optimization experiments of 2 to 5 factors [21,22]. In the present study, CAO derived from red algae was used as a reducing and capping agent to synthesize AuNPs for the first time, and the synthetic conditions of AuNPs were optimized by a RSM. The CAO-AuNPs achieved were characterized and their in vitro antitumor activities on colorectal cancer and breast cancer were also investigated (Figure 1).

## 2. Results and Discussion

### 2.1. Preparation and Characterization of Carrageenan Oligosaccharide (CAO)

**Figure 2:**
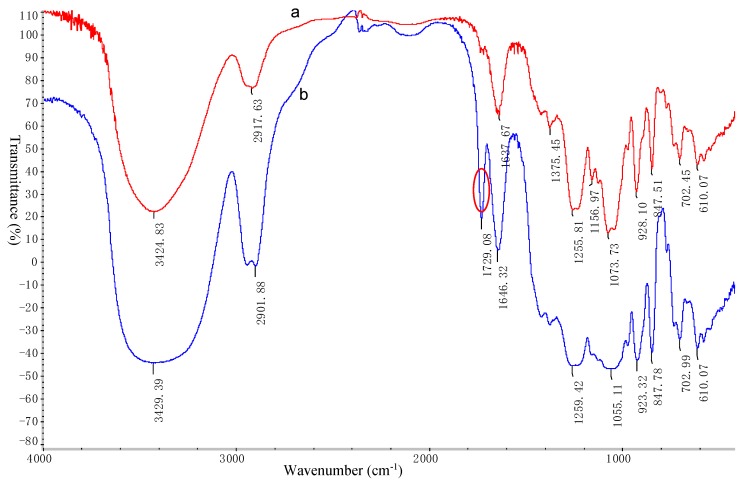
Fourier transform-infrared (FT-IR) spectra of CAO-AuNPs (**a**) and CAO (**b**).

**Figure 3:**
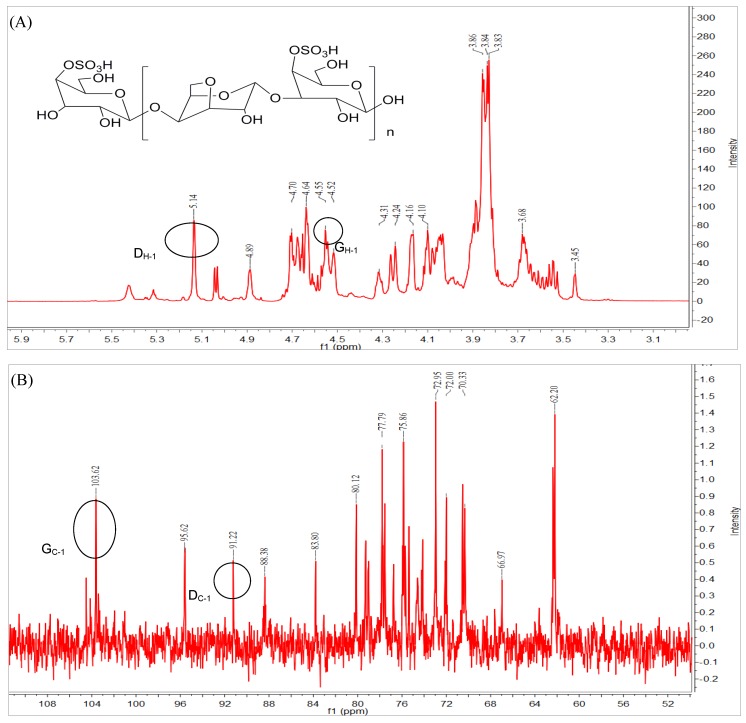
Characterization of CAO: (**A**) proton nuclear magnetic resonance (^1^H NMR) spectra and inset image showed structure of CAO; (**B**) carbon-13 nuclear magnetic resonance (^13^C NMR spectra).

CAO (referred to as kappa-CAO) was prepared by the mild acid hydrolysis of kappa-carrageenan [16], and the mild acid hydrolysis method is simple and effective. By comparing with the peak time of standard CAO (degree of polymerization, dp, 3–11), it was observed that the obtained CAO mainly contain oligosaccharides with degrees of dp3, dp5, dp7 and dp9 as shown in Figure S1. The molecular weight (Mw) of CAO was 1200 Da and the sulfate content of CAO was 22.3%. The structure of CAO was characterized by means of Fourier transform infra-red (FT-IR) spectroscopy, and proton and carbon-13 nuclear magnetic resonance (^1^H-NMR and ^13^C-NMR). The FT-IR spectrum (KBr pellet) of CAO (Figure 2) showed typical absorption bands of the sulfate group (−SO_3_) at 1259 ± 2 cm^−1^, and d-galactose-4-sulphate (residue G) at 848 ± 2 cm^−1^ and 3, 6-anhydro-galactose (residue D) at 923 ± 2 cm^−1^, respectively. The signal at 3429 ± 2 cm^−1^ was attributed to the O–H stretching vibration, and the peak at 2902 ± 2 cm^−1^ was assigned to the C–H stretching vibration. The signal at 1055 ± 2 cm^−1^ was related to the C–O–C skeletal vibration [23,24,25]. As shown in Figure 3A, signals at the range of 3.4 ppm and 4.4 ppm in the ^1^H-NMR spectrum of CAO indicated the presence of sugar ring protons. The signal at δ 4.55 ppm was attributed to the H-1 of *β*-d-galactopyranose (residue G), and the signal at δ 5.14 ppm was assigned to the H-1 of *α*-d-galactopyranose (residue D), which was in agreement with the reported peak positions of CAO [23]. Twelve signals corresponding to the disaccharide repeating unit of CAO were observed in Figure 3B. The signal at 91.22 ppm was assigned to the C-1 of 4-1inked residue D, and the signal of at 103.62 ppm was related to the C-1 of 3-1inked residue G. The region from 60 ppm to 90 ppm was that of the other signals of CAO, as reported previously [23,24,25]. The structure of CAO was determined to be the repeating disaccharide as shown in the inset of Figure 3A.

### 2.2. Optimization of Synthetic Conditions of Gold Nanoparticles (AuNPs) by Response Surface Methodology (RSM)

RSM is an effective statistical method for optimizing complex processes [21,22]. A BBD was arranged at random using Design-Expert 8.0.6 software (as listed in Table 1). On the basis of a pre-experiment, the reaction temperature was set at 50 °C. It was observed that the ultraviolet-visible (UV-Vis) absorbance values of the 17 groups in RSM experiments were mostly around 530 nm in Figure S2. Therefore, the absorbance at 530 nm was chosen as response values (R1).

Analysis of variance (ANOVA) was applied to analyze the RSM model for significance and variance. The quadratic regression model *F*-value of 11.94 with a low probability *p*-value (<0.05) indicated that the model was significant. The “Lack of Fit *F*-value” is used to represent the degree of fitting between the model and the experiment, and the “Lack of Fit *F*-value” of 6.14 (>0.05) implied it was insignificant, which implied that the RSM model fitted the experimental data well [26,27]. For the model fitting, the coefficient of determination (*R*^2^) was 0.9389, indicating that only 6.11% of the total variation was not suitable according to the model. Additionally, the “Adeq Precision” of 9.685 in our experiment indicated that the model with an adequate signal was suitable for the optimal design of this experiment. This RSM model was confirmed to fit the experimental optimization of synthesis of AuNPs well [21,22,27]. The 3D profiles and contour plots of multiple non-linear regression models (Figure S3) were depicted as a graphical interpretation of the experimental levels of each independent variable. It was observed that the contour line was elliptical which indicated the concentration of chloroauric acid and the concentration of CAO had a significant interaction as seen by the absorbance at 530 nm when reaction time remained at the center level in the middle-right plot of Figure S3. The effect of concentration of chloroauric acid (*p* < 0.01) on synthesis of CAO-AuNPs was found to be the most significant. The concentration of CAO also has an influence on the synthesis of CAO-AuNPs [21,22].

Based on the previous research and actual conditions, the factors automatically obtained and selected by Design-Expert 8.0.6 software were fine-tuning for facilitating the operation as follows: the concentration of chloroauric acid was 0.60 mmol/L, the concentration of CAO was 11 mg/mL, and the reaction time was 3 h at a temperature of 50 °C, respectively. The average absorbance of three parallel validation experiments at 530 nm was 1.295, which was well matched with the predicted value with a relative error of 7.48%, indicating that the model was suitable for synthetic condition optimization of CAO-AuNPs [21,27].

### 2.3. Synthesis of CAO-AuNPs

**Figure 4:**
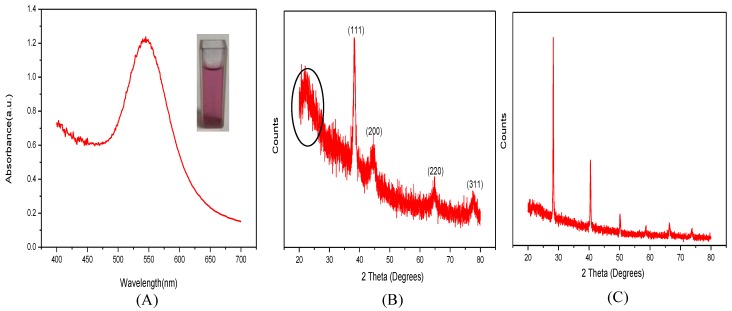
Characterization of CAO-AuNPs: (**A**) ultraviolet-visible (UV-Vis) spectra of CAO-AuNPs and inset photograph showed ruby red color of AuNPs dispersion, (**B**) X-ray diffraction (XRD) pattern of CAO-AuNPs, (**C**) XRD pattern of CAO.

Localized surface plasmon resonance (LSPR) can be defined as light scattering by AuNPs which contributes to the collective oscillation of the electron spectrum in the visible-near infrared (VIS-NIR) region of AuNPs [28]. In Figure 4A, the CAO-AuNPs achieved showed a strong absorption peak at 530 nm with an absorbance value of 1.237, which was due to the LSPR oscillation of AuNPs. The color of the reaction system gradually changed from light yellow to dark ruby red during the reaction, visually indicating the formation of nanoparticles due to the reduction of Au^3+^ into Au^0^ ions in chloroauric acid (as displayed in Figure 4A) [2]. It was revealed that CAO acts as a reducing reagent to obtain CAO-AuNPs. The UV-Vis spectrum of CAO-AuNPs remained unchanged over several weeks when stored at 4 °C in Figure S4, and the solution of CAO-AuNPs was no sign of aggregation, indicating that CAO-AuNPs solution was relatively stable.

### 2.4. Characterization of CAO-AuNPs

The X-ray diffraction (XRD) pattern of CAO and CAO-AuNPs powder by lyophilization that was not altered the polymeric structure [29] is shown in Figure 4B,C. It was observed that four relatively sharp peaks were observed at 2θ degrees, and the peaks of 38.14°, 44.22°, 64.83° and 77.74° correspond to the (111), (200), (220) and (311) face-centered cubic (FCC) structure of crystalline metallic gold, respectively [30,31], indicating that CAO-AuNPs were crystalline (Figure 4B). The resulting crystalline reflections were mainly at 28.26°, 40.44°, 50.12° and 58.70°, suggested that CAO was well-organized crystalline polymer [32]. The XRD differences of CAO and CAO-AuNPs revealed that CAO might be involved in the synthesis of CAO-AuNPs nanocrystals.

The FT-IR spectra of CAO-AuNPs and CAO are shown in Figure 2 (a and b, respectively). Comparing the FT-IR spectrum of CAO with CAO-AuNPs, several functional group signals showed shifts as follows: the peak at 3429 ± 2 cm^−1^ to 3425 ± 2 cm^−1^ were associated with the stretching vibration of O–H, the band observed at 1259 ± 2 cm^−1^ shifting to 1256 ± 2 cm^−1^ was assigned to the −SO^3^^−^ vibration of residue G, and the peak at 1055 ± 2 cm^−1^ varying to 1074 ± 2 cm^−1^ corresponded to the C–O skeletal vibration of residue D from CAO [2,9,22,23]. These results indicated that these functional groups (−OH, −SO^3^^−^, C–O) of CAO might be responsible for the reduction of Au^3+^ and the capping of CAO-AuNPs. It was interesting that the absorption peak at 1729 ± 2 cm^−1^ of CAO disappeared (Figure 2b) compared with CAO-AuNPs (Figure 2a), which indicated that hemiacetal moieties of CAO were involved in the formation of AuNPs [12,33]. In addition, it is worth noting that the change of the finger print region (1200 ± 2–700 ± 2 cm^−1^) peaks, which correspond to stretching vibration of C–OH and C–O–C glycosidic bond, indicated the possible interaction between AuNPs and CAO [20].

**Figure 5:**
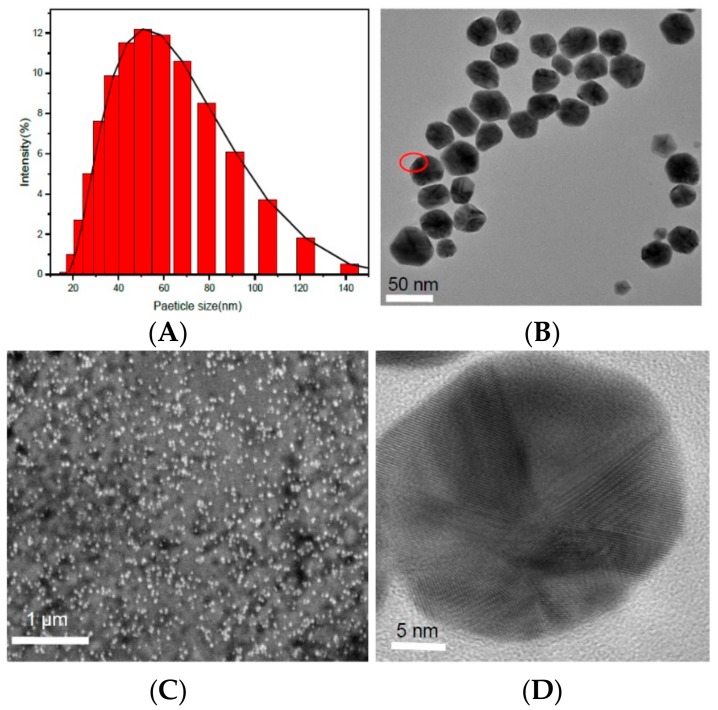
Electron microscopy characterization of CAO-AuNPs: (**A**) particle size distribution graph; (**B**) transmission electron microscope (TEM) image of CAO-AuNPs; (**C**) scanning electron microscope (SEM) image of CAO-AuNPs; (**D**) high-resolution TEM (HRTEM) images of CAO-AuNPs.

The particle average size was 35 ± 8 nm (Figure 5A) with a polydispersity index value of 0.31 ± 0.02 (recorded number, 1375), indicating that CAO-AuNPs showed homogeneous size distribution. The morphological structure of CAO-AuNPs was observed by means of transmission electron microscopy (TEM) (Figure 5B,D) and scanning electron microscopy (SEM) (Figure 5C). Under high-magnification electron microscopy of Figure 5B, most of the CAO-AuNPs were ellipsoidal. At low-magnification scanning electron microscopy of Figure 5C, the CAO-AuNPs showed relatively isolated size distribution (measured amount, 3 mL). In addition, the carbohydrate membrane which was the light-colored transparent part was observed around AuNPs in Figure 5B and Figure S5. The particle average size obtained from TEM, high-resolution TEM (HRTEM) and SEM was smaller than that obtained by Nano-ZS90 Malvern particle size analyzer based on dynamic light scattering (DLC), due to different methods measuring different parameters, and the DLC method was more sensitive to the presence of larger particles. In addition, the forces of interaction between particles in the solution, for example, van der Waals forces, may contribute to larger size determination [34].

The HRTEM result of CAO-AuNPs (Figure 5D) not only showed a clear ellipsoidal shape, but also displayed the distribution of the lattice of AuNPs that was consistent with the FCC structure of gold [10,20,31], which indicated that the obtained AuNPs are crystal structures. The zeta potential (ZP) of CAO-AuNPs was determined to be −20 mV, indicating that the CAO-AuNPs had a high negatively charged surface, which could be beneficial for the delivery of positively charged drugs [2,35]. Moreover, it has been reported that the gold core is a double electron layer structure [36], which may explain the ready reduction of AuNPs by negatively charged carbohydrate. According to the results of TEM (Figure 5B) and FT-IR (Figure 2), it was assumed that the CAO was distributed on the surface of AuNPs, indicating the electrostatic interaction between negatively charged CAO and AuNPs. Therefore, the CAO acted as a stabilizing and capping agent around the surface of AuNPs.

### 2.5. In Vitro Cytotoxicity Study

**Figure 6:**
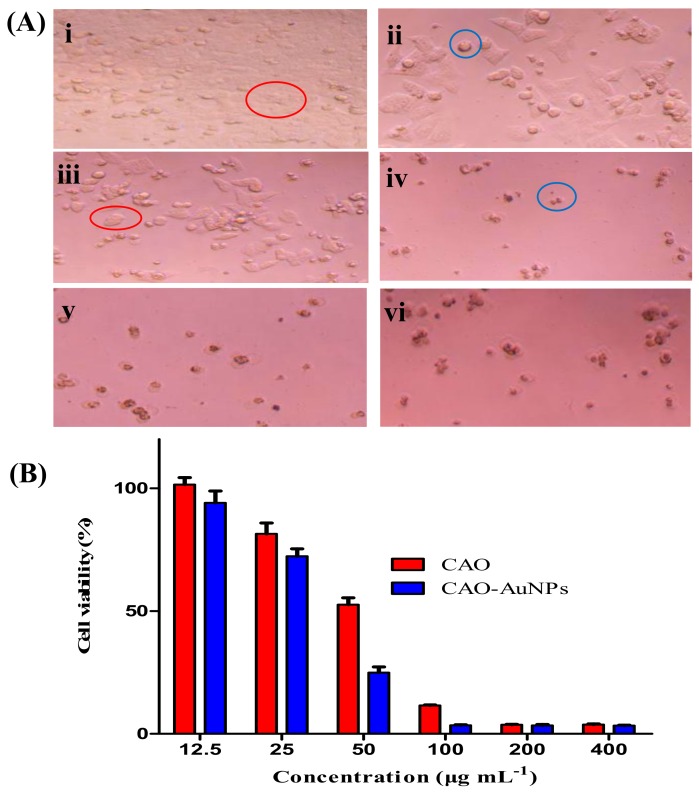
Anti-cancer activity of CAO-AuNPs to HCT-116 cells by the sulforhodamine B (SRB) assay: (**A**) morphological alterations in HCT-116 cells incubated at varied concentration of CAO-AuNPs, and cells in red circle represented living cell while cells in blue circle represented apoptotic/dead cells, (i) control, (ii) 25 μg/mL, (iii) 50 μg/mL, (iv) 100 μg/mL, (v) 200 μg/mL and (vi) 400 μg/mL for 72 h (40× Magnification); (**B**) percentage cell viability of HCT-116 human colon cancer cells after 72 h of treatment with free CAO and CAO-AuNPs (mean ± SD, *n* = 4).

The sulforhodamine B (SRB) assay was relied on the property of SRB, which binds stoichiometrically to proteins under mild acidic conditions and then can be extracted using basic conditions; thus, the amount of bound dye can be used as a proxy for cell mass, which can then be extrapolated to measure cell proliferation and cytotoxicity [37]. The anti-proliferative activities of CAO-AuNPs and free CAO were tested against HCT-116 human colon cancer cells, MDA-MB-231 human breast cancer cells, and HUVEC human umbilical vein endothelial cells by using SRB assay. The results in Figure S6 indicated that the green synthetic CAO-AuNPs were biocompatible in nature. Moreover, the cell morphological changes at different concentrations of CAO-AuNPs for 72 h were shown in Figure 6A. It was observed that the amount of living cells was reduced with increasing concentration of CAO-AuNPs, which were in accordance with the cell viability of CAO-AuNPs. The cytotoxic effects of CAO and CAO-AuNPs showed a concentration-dependent cytotoxicity in the range of 12.5–400 μg/mL, and the cell viability was negatively correlated with concentrations of CAO and CAO-AuNPs (as shown in Figure 6B). The IC_50_ values of free CAO and CAO-AuNPs against HCT-116 cells holded at 49.9 ± 1.6 μg/mL and 34.4 ± 1.7 μg/mL in 72 h, respectively [38]. Similarly, the IC_50_ values of free CAO and CAO-AuNPs to MDA-MB-231 cells were 164.2 ± 1.8 μg/mL and 129.2 ± 1.7 μg/mL, respectively. The differences of IC_50_ values among free CAO and CAO-AuNPs on cancer cells indicated that the CAO-AuNPs not only possessed stronger cytotoxicity to cancer cells than that of free CAO, but also exhibited greater effectiveness to HCT-116 cells than that to MDA-MB-231 cells via galactose receptor-mediated endocytosis [39].

**Figure 7:**
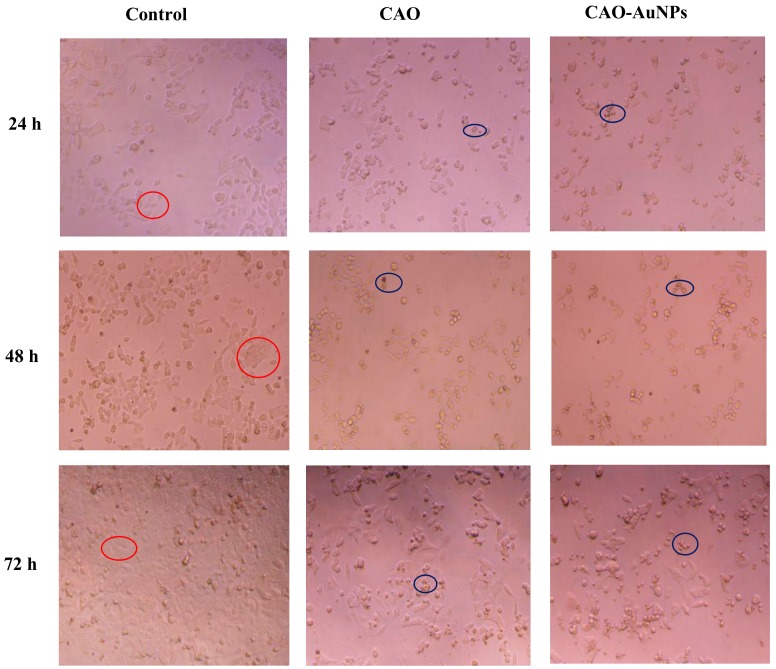
Morphological variations in HCT-116 human colon cancer cells incubated with 50 μg/mL of CAO and CAO-AuNPs for 24 h, 48 h and 72 h, and cells in red circle represented living cell while cells in blue circle represented apoptotic/dead cells, as assessed by a CX41 microscope (40× Magnification).

The cytotoxicity of CAO-AuNPs at 50 μg/mL against HCT-116 cells at 24 h, 48 h and 72 h is shown in Figure 7. It was observed that the cells proliferated in a time-dependent way in the control. After treatment of CAO and CAO-AuNPs at 24 h, 48 h and 72 h, respectively, the amount of living cells was reduced (Figure 7), indicating that the proliferation of cells was inhibited. In addition, CAO-AuNPs induced more mortality of the HCT-116 cells than that by CAO, indicated that CAO-AuNPs had stronger cytotoxicity than CAO against HCT-116 cells, which was in good agreement with the SRB assay. The strong cytotoxicity of CAO-AuNPs might be related to the accumulation of AuNPs in target cells by endocytosis [40]. Furthermore, the attachment of CAO around AuNPs led to an enhanced anticancer activity compared with free CAO, which might be related to the cell targeting of AuNPs. To our knowledge, CAO with low Mw has been reported to enhance its cytotoxicity and anticancer activity by increasing macrophage phagocytosis [41,42]. In addition, the negatively charged AuNPs were easily recognized by macrophages than positively or neutrally charged AuNPs [40,42]. In summary, the CAO-AuNPs showed certain cytotoxicity on cancer cells.

## 3. Materials and Methods

### 3.1. Materials and Reagents

Carrageenan derived from marine red algae was provided by Qingdao Marine Biomedical Research Institute (Qingdao, China). Gold (III) chloride trihydrate (HAuCl_4_·3H_2_O) was purchased from Sigma-Aldrich (St. Louis, MO, USA). Water used throughout the experiments was double distilled and purified on a Milli-Q system (Millipore Inc., Milford, MA, USA). McCOY’S 5A-MEDIUM (5A) medium was purchased from HyClone (Logan, UT, USA). Dulbecco’s modified eagle medium (DMEM), L-glutamine, 100 U/mL penicillin and 100 g/mL streptomycin were purchased from Gino Biopharmaceutical Technology Co., Ltd. (Hangzhou, China). Fetal bovine serum (FBS) was purchased from ExCell Bio (Shanghai, China). All other chemicals and solvents used were of analytical grade unless otherwise specified.

### 3.2. Preparation of Carrageenan Oligosaccharide

CAO was prepared by mild acid hydrolysis of kappa-carrageenan which fractionated with KCl from carrageenan. Briefly, kappa-carrageenan (10 g) was hydrolyzed in a solution of 0.1 mol/L HCl (1 L) at 65 °C for 1 h. The partial hydrolysis was stopped by neutralization with 1 mol/L NaOH, and the hydrolysate was desalted on a Sephadex G10 column (1.6 cm × 60 cm; Amersham Biosciences, Uppsala, Sweden) [16]. The eluent was collected and concentrated by a rotary evaporator (Heidolph, Schwabach, Germany), and then lyophilized to yield a pale yellow solid of CAO. The composition of CAO was determined by a gel filtration chromatography on a Shodex OHpak SB 802.5 HQ column (8.0 mm × 300 mm, 6 μm; Showa Denko K. K., Kawasaki, Japan). The sulfate content of CAO was determined by a SH-AC-3 anion column (9 µm, 4 mm × 25 mm; Qingdao ShengHan Chromatograph Technology Co., Ltd., Qingdao, China) on a Shine CIC-100 ion chromatograph (Qingdao, China) [43]. The Mw of CAO was determined by high-performance liquid chromatography (HPLC) coupled with refractive index detector (Agilent Technologies, Wilmington, DE, USA) with a column of TSKgel G3000PWXL (TOSOH, Tokyo, Japan). Aqueous Na_2_SO_4_ solution (0.1 mol/L) was used as the mobile phase and the flow rate was 0.5 mL/min. The temperature of the column was kept at 35 °C. Dextrans were used as standards to calibrate the column [44].

### 3.3. Fourier Transform-Infrared (FT-IR) and Nuclear Magnetic Resonance (NMR) Analysis of Carrageenan Oligosaccharide

The FT-IR spectra of CAO and CAO-AuNPs power that was lyophilized in a FD-1C-50-type freeze dryer (Beijing Bo Medical Experimental Instrument Co., Ltd., Beijing, China) were recorded on a Nexus 470 spectrophotometer (Nicolet, San Diego, CA, USA) as KBr pellets over a wavelength range of 400–4000 cm^−1^ at a resolution of 2 cm^−1^. For NMR analysis, CAO (25 mg) which was dissolved in D_2_O (99.96 atom%) was freezed by a −40 °C refrigerator, and was lyophilized in a FD-1C-50-type freeze dryer (Beijing Bo Medical Experimental Instrument Co., Ltd., China) three times to prepare deuterated CAO. ^1^H-NMR (500 MHz) and ^13^C-NMR (125 MHz) were recorded at 20 °C on an Agilent DD2-500 instrument (Agilent Technologies, Santa Clara, CA, USA). Chemical shifts were measured relative to internal acetone-d6 at 2.22 ppm (^1^H) and 30.89 ppm (^13^C).

### 3.4. Optimization of Reaction Condition for Synthesis of AuNPs by RSM

Through preliminary experiments, the influence factors of preparation of CAO-AuNPs were determined as follows: concentration of gold chloride acid (A), reaction time (B) and concentration of CAO (C). Then the reaction conditions for synthesis of AuNPs were further optimized by RSM. A BBD design [21,22] was performed on three independent variables (A, B, C) at three levels. The three levels of factor A, B and C were 0.1, 0.5, 1.0 mmol/L; 3 h, 4 h, 5 h and 40, 50, 60 mg/5 mL, respectively. The complete design consisted of 17 experiments as presented at random order in Table 1.

### 3.5. Synthesis of CAO-AuNPs

On the basis of RSM optimization, the experimental factors were fine-tuning for the convenience of operation. Briefly, CAO (0.11 g) was added to 10 mL aqueous solution of 6 × 10^−4^ mol/L HAuCl_4_·3H_2_O, and the solution was stirred at 50 °C for 3 h, which resulted in a dark ruby red color, indicating the formation of CAO-AuNPs. The CAO-AuNPs dispersion was dialyzed against distilled water using a 14 kDa cut-off dialysis membrane to remove ionic impurities and unreacted reducing oligosaccharides. The suspension of CAO-AuNPs was freezed for about 2 h by a −40 °C refrigerator. Then the freezed CAO-AuNPs was lyophilized by a FD-1C-50-type freeze dryer to prepare CAO-AuNPs powder.

### 3.6. Characterization of CAO-AuNPs

The morphology of CAO-AuNPs prepared by dialysis treatment was evaluated using a JEM-2100EX TEM, HRTEM (Jeol, Tokyo, Japan), and a JSM-6700F SEM (Jeol, Tokyo, Japan). XRD of CAO-AuNPs powder was undertaken using a D-MAX 2500/PC XRD diffractometer (Rigaku, Tokyo, Japan) with Cu Kα radiation (*λ* = 0.1518 nm). The particle size and ZP of CAO-AuNPs obtained by dialysis treatment were determined by using a Nano-ZS90 Malvern particle size analyzer (Malvern Instruments Ltd., Malvern, UK) at a scattering angle of 90° and at room temperature. UV-Vis spectrum analysis of CAO-AuNPs was performed on a UV-Vis spectrophotometer 2800 (Shimadzu, Kyoto, Japan) with quartz cell at a resolution of 1 nm.

### 3.7. In Vitro Cytotoxicity Study

#### 3.7.1. Cell Viability Assay

Human breast cancer cells (MDA-MB-231), human colon cancer cells (HCT-116) and human umbilical vein endothelial cells (HUVEC) were cultured at 37 °C in an atmosphere of 5% CO_2_ in an incubator. The cells were plated in 96-well culture plates at a density of 4 × 10^3^ per well, and then incubated for 24 h to allow cell adhesion. Next day, the cells were treated at different doses of CAO and CAO-AuNPs (12.5, 25, 50, 100, 200, 400 μg/mL) for 72 h. Then, cells were fixed with cold trichloroacetic acid (TCA, 50%) at 4 °C to stand for 1 h. After incubation, the cells were washed four times by slow-running tap water. Then the SRB solution was added to each well at room temperature for 1 h and subsequently the plates were quickly rinsed four times with acetic acid (1%) to remove unbound dye. After SRB staining, 150 μL/well Tris solution was added in the cultures and the optical density was measured at 540 nm in a multi-well enzyme-linked immunosorbent assay (ELISA) plate reader (Molecular Devices, San Diego, CA, USA). The control group was referred to that the cells were without treated by CAO and CAO-AuNPs, and the other culture conditions were the same as the sample group. Optical density (OD) value was transformed into the percentage of cell viability using the following formula:

Cell viability (%) = (OD value of samples/OD value of controls) × 100(1)

#### 3.7.2. Cell Morphology Observation

MDA-MB-231 and HCT-116 cells were treated with various concentrations of CAO and CAO-AuNPs and incubated for 24 h, 48 h and 72 h at 37 °C under a 5% CO_2_ atmosphere. After incubation, the morphological variation of cells was observed by a CX41 microscope (Olympus, Tokyo, Japan).

### 3.8. Statistical Analysis

Analysis of variance (ANOVA) and multiple regression analysis were conducted for fitting the model using the Box–Behnken design. The experimental data on the optimization for synthetic conditions of CAO-AuNPs were analyzed by Design-Expert 8.0.6 software (Stat-Ease, Inc., Minneapolis, MN, USA). Data acquired from characterization of CAO-AuNPs were analyzed by means of Origin 8.0 (OriginLab Corporation, Northampton, MA, USA). All experiments data in cytotoxicity study were performed in four times and presented as mean ± standard deviation (SD). Data obtained were analyzed using GraphPad Prism 5.0 (GraphPad Software, San Diego, CA, USA) and the one-way ANOVA model of SPSS 20.0 (IBM, Armonk, NY, USA).

## 4. Conclusions

CAO derived from red algae served a dual role as both the reducing and stabilising agents to synthesize AuNPs for the first time. This synthetic method is green, efficient and eco-friendly, and the reaction conditions were optimized by RSM. The biocompatible and stable CAO-AuNPs obtained were proved to be crystal, monodispersed and mostly ellipsoidal by means of XRD, TEM, SEM and a particle analyzer. Furthermore, the CAO-AuNPs exhibited significant activities against HCT-116 and MDA-MB-231 cells. This study provides references for the green preparation of AuNPs by marine carbohydrates in the field of nanomaterials. Furthermore, the CAO-AuNPs nanosystem shows a promising prospect of application in nanomaterial-based drug delivery, and it could be worthy of further investigation of CAO-AuNPs as a nanocarrier for anti-cancer drugs.

## Figures and Tables

**Table 1:** Box–Behnken experimental design and results.

Std Order *	Run Order	Factor1 A:Concentration of Gold Chloride Acid (mmol/L)	Factor2 B:Reaction Time (h)	Factor3 C:Concentration of CAO (mg/5 mL)	Absorbance at 530 nm (R1)
11	1	0.5	3	60	1.311
9	2	0.5	3	40	1.033
13	3	0.5	4	50	1.044
1	4	0.1	3	50	0.200
16	5	0.5	4	50	1.139
12	6	0.5	5	60	1.163
3	7	0.1	5	50	0.188
10	8	0.5	5	40	0.993
15	9	0.5	4	50	1.140
7	10	0.1	4	60	0.195
17	11	0.5	4	50	1.142
14	12	0.5	4	50	1.305
4	13	1.0	5	50	0.872
5	14	0.1	4	40	0.180
8	15	1.0	4	60	0.550
6	16	1.0	4	40	0.060
2	17	1.0	3	50	0.882

* Std order is the standard order that Design-Expert 8.0.6 software calculated according to experimental data.

## Supplementary Materials

The following are available online at http://www.mdpi.com/1660-3397/16/8/277/s1, Figure S1: The elution profile of CAO by a Shodex OHpak SB-802.5 HQ column; Figure S2: UV-Vis spectra of the 17 groups in RSM experiments; Figure S3: Response surface plot and contour plot of effects on synthetic conditions of AuNPs; Figure S4: UV-Vis spectra of CAO-AuNPs of four weeks stability at different times; Figure S5: Electron microscopy characterization of CAO-AuNPs, SEM image of CAO-AuNPs; Figure S6: Cell viability assay of CAO-AuNP on HUVEC cells at different concentrations (6.25–200.00 μg/mL) for 72 h by the SRB.
