# Supplementary material for: Green Synthesis of Gold Nanoparticles Using Carrageenan Oligosaccharide and Their In Vitro Antitumor Activity
Source: Mar Drugs. 2018 Aug 7;16(8):277. doi: 10.3390/md16080277 (PMC6117638; doi:10.3390/md16080277)
Supplement: Supplementary file 1 [file marinedrugs-16-00277-s001.pdf]

Figure S1

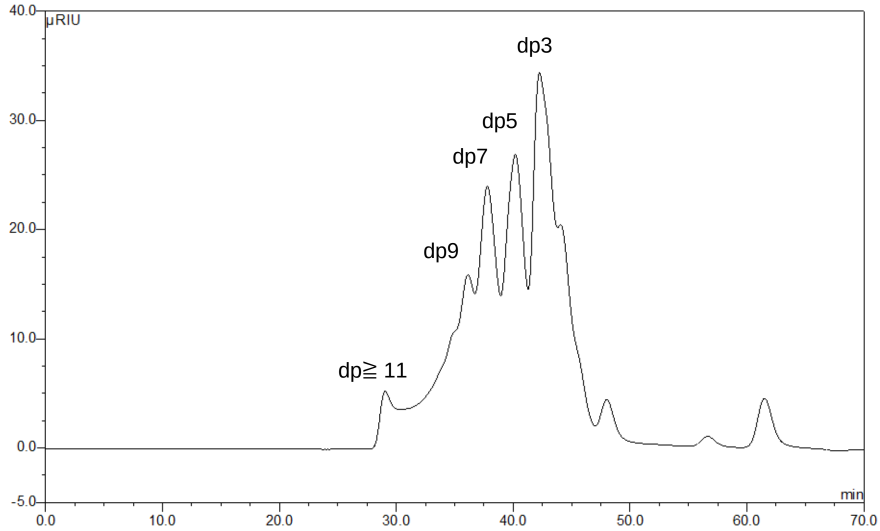

Figure S1. The elution profile of CAO by a Shodex OHpak SB-802.5 HQ column.

Figure S2

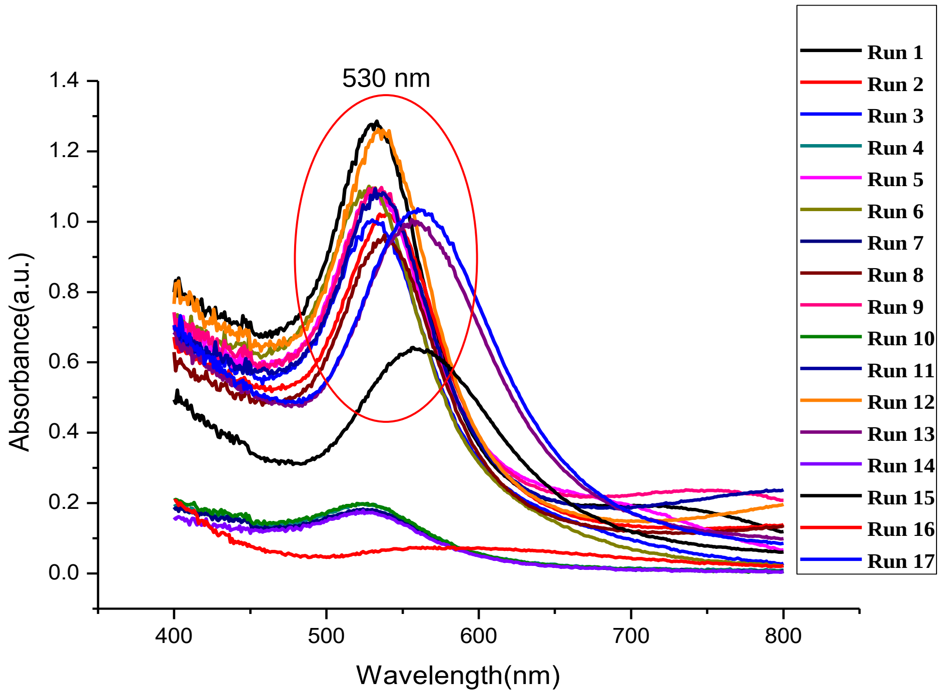

Figure S2. UV-vis spectra of the 17 groups in RSM experiments.

Figure S3

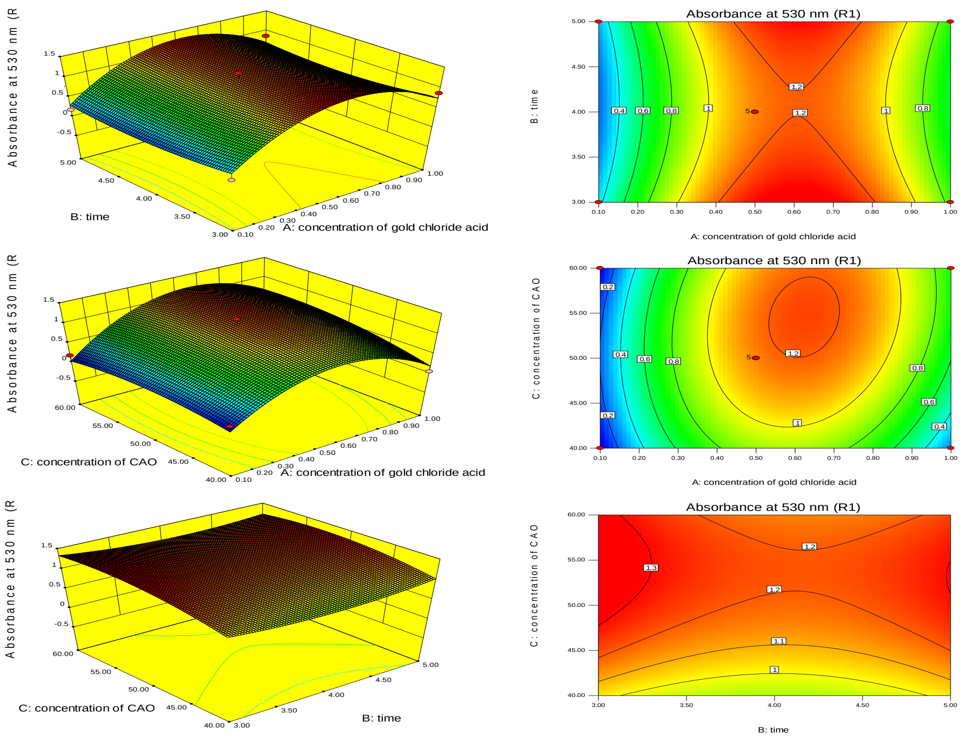

Figure S3. Response surface plot and contour plot of effects on synthetic conditions of AuNPs.

Figure S4

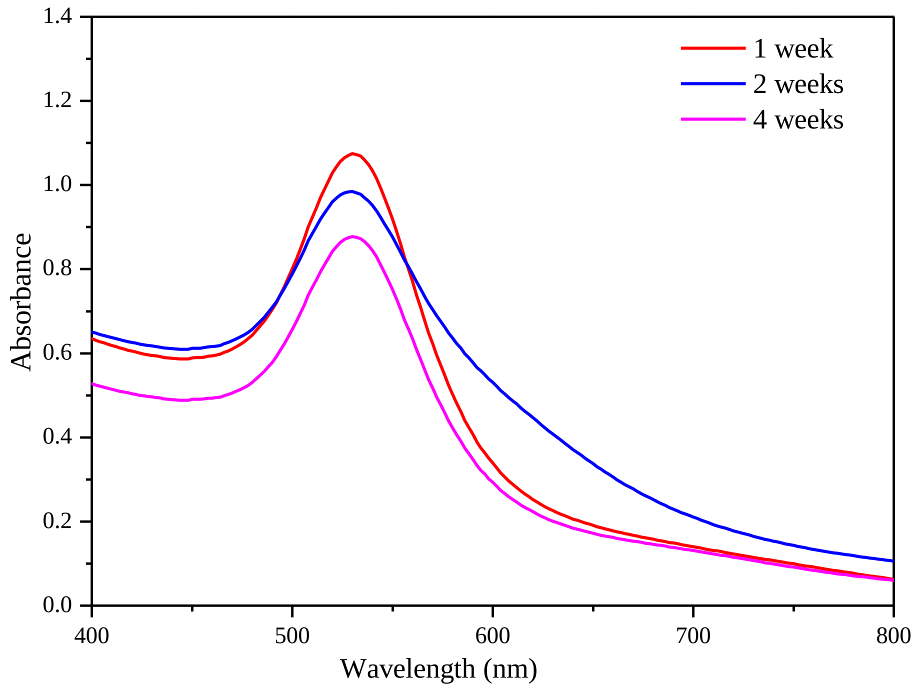

Figure S4. UV-vis spectra of CAO-AuNPs of four weeks stability at different times.

Figure S5

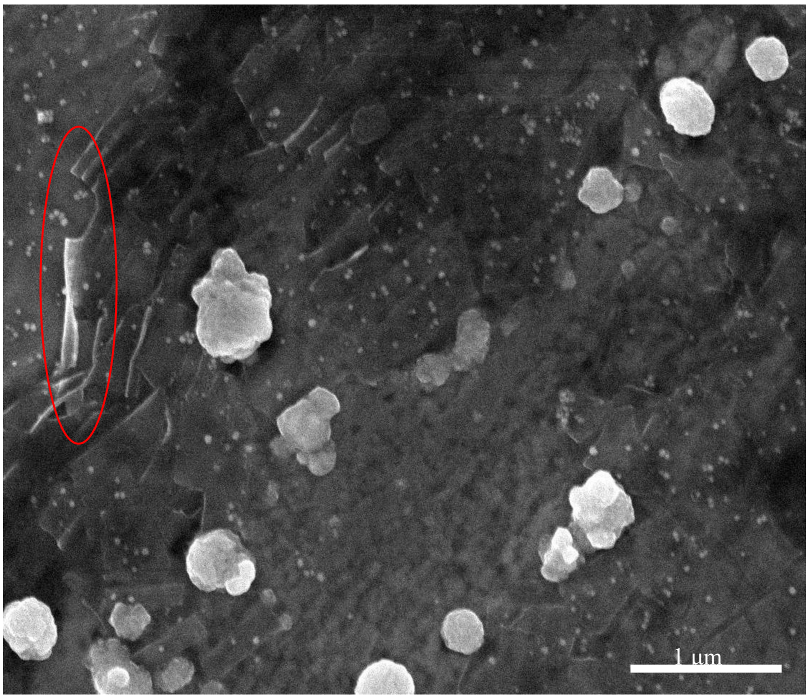

Figure S5. Electron microscopy characterization of CAO-AuNPs, SEM image of CAO-AuNPs.

Figure S6

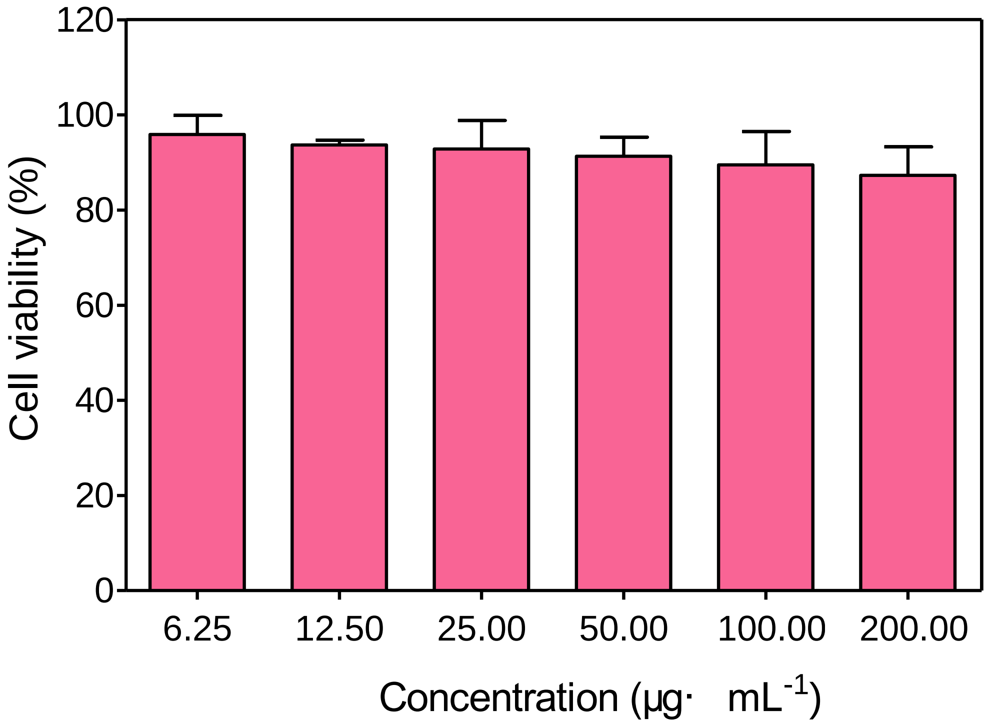

Figure S6. Cell viability assay of CAO-AuNP on HUVEC cells at different concentrations (6.25-200.00 μg/mL) for 72 h by the SRB.
